# Locally induced shockwaves for selective perforation of cargo loaded lipid vesicles with temporal and spatial control†

**DOI:** 10.1039/d3ra03988a

**Published:** 2023-08-21

**Authors:** Jure Derganc, Špela Zemljič-Jokhadar, Boris Majaron, Gašper Kokot

**Affiliations:** a Institute of Biophysics, Faculty of Medicine, University of Ljubljana Ljubljana Slovenia gasper.kokot@ijs.si; b Jožef Stefan Institute Ljubljana Slovenia; c Faculty of Mathematics and Physics, University of Ljubljana Ljubljana Slovenia

## Abstract

Controlled poration of lipid membranes is crucial for numerous biomimetic applications such as targeted drug delivery. Although several chemical and physical mechanisms have been proposed for the poration of synthetic membranes, achieving good temporal and spatial control remains a challenge. In this study, we introduce a novel method for membrane poration that utilizes the mechanical shockwave generated by the photo-acoustic effect, which occurs when an optically opaque microparticle is illuminated by a near-infrared laser of optical tweezers. We show that the shockwave effectively porates membranes of giant unilamellar vesicles in close proximity to the microparticle without damaging nearby cells, which is a desirable outcome for potential targeted drug delivery. The poration effect is nonspecific and operates on both liquid and gel phase membranes. Since the photo-acoustic effect can be triggered by standard optical tweezers, this method holds broad applicability in various experimental settings within the field of soft matter research.

## Abstract 2

Controlled poration of lipid membranes is crucial for numerous biomimetic applications such as targeted drug delivery.

## Graphical abstract

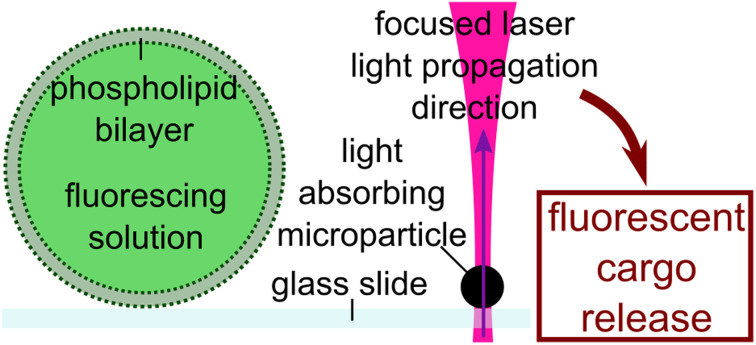

## 1 Introduction

Regulated transmembrane transport, a fundamental characteristic of living organisms, remains a formidable challenge in developing artificial biomimetic systems. Numerous strategies have been devised to achieve controlled membrane permeabilization, particularly for targeted drug delivery^1^ and bottom-up synthetic biology.^2^ Some approaches are based on meticulously engineered membranes that respond to specific chemical stimuli such as pH changes,^3^ or physical triggers like temperature,^4^ light,^5,6^ or magnetic field.^7^ Another category of methods induces membrane rupture by applying lateral mechanical stress, utilizing, for example, electric fields or mechanical waves. These approaches offer advantages such as not being restricted to specially designed membranes and avoiding introducing potentially harmful chemicals into the system. Two such methods, electroporation and sonoporation, have thus become standard laboratory procedures. However, these two methods act in bulk and lack the ability to target with a single cell precision.^8^

This study presents a novel approach for nonspecific lipid membrane permeabilization that offers excellent temporal and spatial control. The method exploits a shockwave triggered by photo-acoustic effect, which occurs after illuminating an opaque microparticle with a focused near-infrared (NIR) laser. Importantly, we show that a laser integrated in standard optical tweezers has suitable power to induce this effect. The impact of the shockwave is confined to membranes near the microparticle, so the technique can be readily used in systems comprising living cells.

## 2 Experimental

### 2.1 Vesicles and cells

Giant unilamellar vesicles (GUVs) were prepared using the standard electroformation in a sucrose solution containing Alexa 488 fluorescent dye (Invitrogen, A10436) at a concentration of 5 μmol L^−1^.^9^ Subsequently, the GUVs were transferred to an isomolar glucose solution to ensure their stability and to restrict fluorescent dye exclusively to their interior. The GUVs were prepared with either egg yolk phosphatidylcholine (EPC, Avanti Polar Lipids, 840051P), which is in liquid phase, or dipalmitoylphosphatidylcholine (DPPC, Avanti Polar Lipids, 850355P), which is in gel phase at room temperature. The EPC GUVs were prepared at room temperature and the DPPC GUVs at 40 °C, because it is in the liquid phase at that temperature. To enable the binding of streptavidin-coated microparticles to the GUV membrane, the GUVs were prepared with 0.1% of biotinylated lipids (DSPE-PEG-2000 Biotin, Avanti Polar Lipids, 880129P).

To evaluate the effect of shockwaves on living cells, we used HUVEC (ATTC) cells, grown in Advanced MEM (Thermofisher) with 5% fetal calf serum (FBS) and streptomycin/penicillin antibiotics (both Thermofisher). Prior to the experiments, the cells were labelled with fluorescent marker calcein AM (Thermofisher, 10 μmol l^−1^) for 20 minutes and subsequently washed with the growing medium. Once this marker is internalized by the cells, intracellular esterases cleave the AM ester group, yielding the membrane-impermeable calcein, which can only leak out through membrane pores, *e.g.*, during apoptosis.

### 2.2 Experimental setup

Imaging was conducted using an inverted microscope (Nikon Ti-U), equipped with a water immersion objective (Nikon Plan APO VC, 60×, NA = 1.2) and LED EPI illumination (CoolLED pE-300), enabling rapid alternation between transmitted and EPI illumination modalities. The transmitted mode was employed to assess the integrity of GUV membranes, while fluorescence illumination served to quantify the cargo content within the GUVs. Optical tweezers (Tweez, Aresis d.o.o., Slovenia, Nd:YAG laser, *λ* = 1064 nm) with acousto-optic deflectors (AOD) for beam steering were integrated into the microscope. The laser power was measured by a power meter Coherent PM USB PS10.

Shockwaves were triggered by focusing the laser on optically opaque polystyrene microparticles (Dynabeads M-270 Streptavidin, ThermoFisher Scientific, nominal diameter *d*_p_ = 2.8 μm), which had a streptavidin coating to enable binding to biotin molecules in the GUV membrane. A high-speed camera (Photron SA-Z type 2100K-M-64GB) was used for evaluation of the microparticle response to laser illumination.

## 3 Results and discussion

**Fig. 1:**
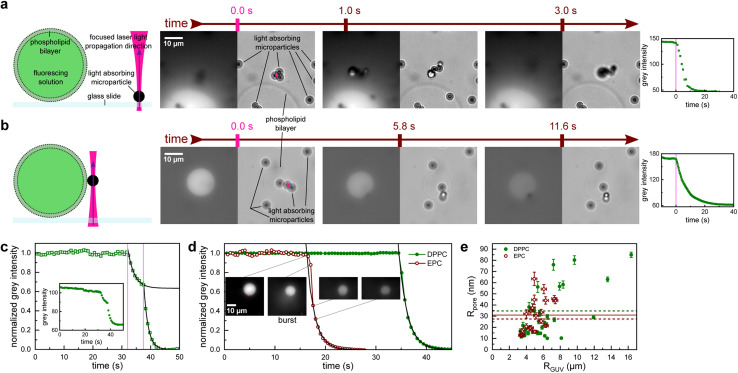
Poration of giant unilamellar vesicles (GUVs) by focusing a near-infrared laser to a nearby microparticle. (a) A poration experiment when the microparticle is resting on a glass slide next to a GUV and (b) when the microparticle is attached to the GUV membrane. After the particle is illuminated by the laser (pink dot, the size matches the laser beam waist diameter) at *t* = 0, the nearby GUV starts leaking the fluorescent solution. Subsequent sequences of brightfield (right) and EPI fluorescence (left) images are shown along with the measured decrease of the fluorescence intensity in the GUV (far right). (c) Fluorescence intensity in a rare case of incomplete leakage after the first illumination (first vertical pink line). The leakage was then reinitiated by a second illumination (second vertical pink line). Fluorescence decay time was similar for the first and second leakage. Inset: non-normalized fluorescence signal shows an initial plateau that is slowly linearly dropping. This was due to photobleaching and was accounted for in further analysis. (d) Fluorescence intensity after illumination-induced poration for an EPC vesicle (red empty circles) and a DPPC vesicle (green full circles). The EPC membrane poration is accompanied by a slight burst (see ESI Video 1†) that leads to a decrease in the vesicle radius (see inset images; the scale bar is the same for all of them). The burst is also responsible for the initial deviation from the exponential decay (black line). For (c) and (d) black lines are an exponential least squares fit. (e) Comparison of the effective pore size *R*_pore_ and the GUV size *R*_GUV_. The mean *R*_pore_ values for DPPC (full green line) and EPC (full red line) almost coincide. The dashed lines represent the standard error of the mean.

To demonstrate the proposed method, we conducted experiments which involved four main components (see the sketch in Fig. 1a): a giant unilamellar vesicle (GUV) serving as a cargo carrier, fluorescent dye as cargo, an optically opaque microparticle and a focused NIR laser employed to generate a shockwave *via* the photo-acoustic effect within the microparticle. A water immersion objective with a large NA provided the laser beam with a waist diameter *w*_0_ = 0.4 μm at the sample plane, which is significantly smaller than microparticle diameter (*d*_p_ = 2.8 μm). Therefore, all the laser light was delivered to a single microparticle (Fig. 1 pink dot). The optical tweezers use acousto-optic deflector (AOD) shutter and steering, which provides the precision to target a selected particle at a specific location at a particular time. Thus, with the help of AOD, we could easily and reliably illuminate a single microparticle on demand. The microparticles are made of polystyrene, they are smooth and spherical, and strongly absorb the laser light due to iron oxide nanoparticles dispersed within their volume.^10^

Upon focusing the laser on a microparticle, a rapid displacement of the particle ensued, accompanied by a decrease in fluorescence signal within an adjacent GUV, indicating the initiation of cargo leakage from the vesicle. This effect was observed if the particle was initially adsorbed to the glass slide in proximity to the GUV (Fig. 1a and S1†), as well as when the particle was bound to the GUV membrane (Fig. 1b). Simultaneous recording of fluorescence and brightfield images confirmed the absence of visible damage to the GUV while exhibiting a decay in fluorescence intensity. This observation suggests the presence of perforations in the phospholipid bilayer, albeit not to the extent that would compromise its integrity. Notably, only particles located within a close proximity (<7 μm) to the GUV triggered the observed fluorescence decay. Laser powers for these experiments were in the range 1 mW < *P* < 5 mW.

In a typical experiment, the fluorescence intensity within the GUV remained stable, except for photobleaching (see inset Fig. 1c), which was accounted for in the subsequent analysis. When the laser was directed onto a nearby microparticle (examples in Fig. 1), the fluorescence intensity exhibited an exponential decay as the cargo was released from the vesicle with a characteristic time constant *τ* (black lines in Fig. 1), which was on the order of several seconds. In some vesicles, the leakage ceased before all the cargo was released (Fig. 1c), which could indicate the closure of the pores over time or obstruction of the pore by the microparticle. In such cases, the illumination-induced poration could be readily repeated, as denoted by the subsequent vertical line in Fig. 1c.

The pore opening and closing process in lipid membranes is a complex interplay of many membrane parameters, such as line tension and viscosity.^11^ In general, lipid membranes can be composed of liquid and gel phases with markedly different mechanical properties. It was shown that the membrane state significantly influences GUV electroporation.^12^ To investigate the potential impact of membrane composition on illumination-induced poration, we analyzed the leakage from vesicles made from two phospholipids: egg yolk phosphatidylcholine (EPC), which is in liquid phase, and dipalmitoylphosphatidylcholine (DPPC), which is in gel phase at room temperature. The only noticeable difference between EPC and DPPC vesicles was in the initial moments of the cargo release. EPC consistently showed a burst accompanied by a decrease in the GUV radius (see snapshots in Fig. 1d and ESI Video 1†). An average ratio of *R*_GUV_ after *vs.* before the burst was 0.89 ± 0.02. Consequently, the decay times for EPC GUVs were obtained from the normalized intensity after the burst was over. Conversely, cargo release from DPPC vesicles was uneventful and faithfully followed the exponential curve.

The experimental resolution did not permit the estimation of the pore radius directly from the images of leaking vesicles. Also, the model of pore closing^11^ has too many interdependent open parameters, so fitting the model parameters to the leakage curves proved futile. We have therefore employed a simplified model of a cargo leaking through a single cylindrical pore with an effective radius *R*_pore_ and length *l*_pore_ (membrane thickness). Assuming the concentration of the fluorescent dye outside the vesicles stays zero and solving Fick's diffusion equation yields exponential concentration time dependence *c* = *c*_0_ exp(−*t*/*τ*), where *c*_0_ is the initial concentration inside the vesicle and *τ* is the characteristic leaking time, which has a simple relation to the effective pore radius:1
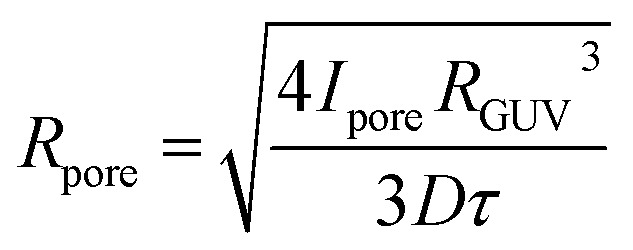
where *D* = 435 μm^2^ s^−1^ is the diffusion constant for Alexa 488 (ref. 13) and *R*_GUV_ is the radius of the GUV, which was determined by fitting a circle to each automatically thresholded fluorescence image. Because there is a linear relation between the concentration of the cargo *c* and its corresponding fluorescence intensity *I*,^14^*c*/*c*_0_ = *I*/*I*_0_ = exp(−*t*/*τ*), the characteristic time *τ* could be obtained by fitting an exponential function to the fluorescence decay using the standard Levenberg Marquart algorithm, where *I*_0_ was determined from the fluorescence intensity at the moment of the laser trigger.

We found that the effective pore radius was *R*_pore_ = (31.1 ± 3.4) nm for EPC GUVs (mean ± SEM of 20 measurements), and *R*_pore_ = (30.9 ± 4.0) nm for DPPC GUVs (mean ± SEM of 31 measurements). Remarkably, both pore radii almost coincide (the comparison of means using a two-tailed *t*-test yields a *p*-value of 0.93), suggesting that the damage from the shockwave is similar for a GUV membrane in a liquid phase (as with EPC, Fig. 1e red empty circles) and a gel phase (as with DPPC, Fig. 1e green full circles). The effective pore radius *R*_pore_ is large enough to accommodate even the largest drug molecules, such as monoclonal antibodies, which have a diameter of about 10 nm.^15^ Interestingly, the pores observed in our experiments did not resemble “macropores” obtained by electroporation that are several micrometers in diameter and relax with relaxation times of less than 1 s.^12,16^

**Fig. 2:**
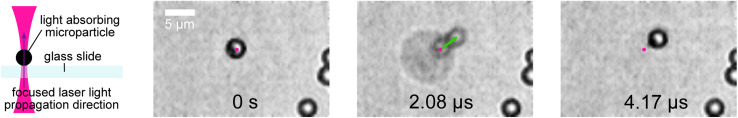
An illumination event recorded by a high-speed camera. The position of the laser beam is marked with a pink dot (the diameter matches the laser beam waist diameter). The evidence of a shockwave shows at 2.08 μs. This image is blurred due to the rapid shockwave expansion (large circular region). The apparent double particle image is a consequence of a fast particle displacement within the same exposure (as indicated by the green arrow). The laser beam remains fixed at the initial spot and falls outside the particle after its displacement. The shockwave generation is much faster than the camera frame rate. The scale bar is the same for all the images.

In order to elucidate the mechanism of the observed GUV poration, we recorded an illumination event of a microparticle bound to a glass substrate with a high-speed camera. Within the first frame after the illumination, the microparticle moved away (Fig. 2, green arrow) but was not visibly damaged. Assuming that microparticles are mostly polystyrene and spherical, we calculated the thermal relaxation time‡‡We calculate the thermal relaxation time with the formula for spherical relaxation: *d*_p_^2^/(27*D*), where *D* is the thermal diffusivity of polystyrene: *D* = 0.122 mm^2^ s^−1^.^18^ to be 2.2 μs and the stress relaxation time§§We calculate the stress relaxation time with the formula: *d*_p_/(2*v*_c_), where *v*_c_ is the speed of sound in polystyrene: *v*_c_ = 2350 m s^−1^.^19^ to be 0.6 ns. These conditions are known to induce the photo-acoustic effect and a subsequent shockwave.^17^ Indeed, a shockwave around the microparticle is visible in the second image of Fig. 2 as a dark circle with a white halo (area of decreased density due to the advancing shockwave front, thus providing a mismatch in the carrier solution refractive index, Fig. 2b, snapshot at 2.08 μs). The blurred image of the shockwave indicates that its front moves rapidly compared to the camera exposure time. Another telltale sign of rapid motion comes from the illuminated particle on the same image: the frame with the shockwave seemingly comprises two particles, but the frames both before and after show only one particle. In fact, the double-particle image represents the same particle at two positions during the same exposure. Since the contrast at the two positions is comparable, we estimate the particle spent about half the exposure time at the first position and then moved to the second position almost instantaneously (note the absence of a noticeable blur between the two positions). Repeated experiments revealed that illuminated particles displace in random directions, including both lateral and vertical movements, showing that the starting point of the shockwave deviates randomly from the particle's center. Based on these observations, we propose that the observed membrane poration is induced by the shockwave generated from the photo-acoustic effect triggered by the laser illumination of the microparticle. As the shockwave propagates spherically, it losses intensity with the square of the distance from the origin. Consequently, only microparticles in close proximity to a GUV are capable of inducing poration. Hence, this method provides excellent temporal and spatial control over the GUV cargo release.

Of note, if the particles were illuminated with a low laser power (*P* < 1 mW or power density 1.4 MW cm^−2^ at *w*_0_) we observed only the normal effects of optical tweezers on microparticles that strongly absorb the incident laser light – a freely floating particle was laterally attracted to the laser focus and got pushed out of focus along the axial direction by radiation pressure. If the particles were bound to the glass substrate, low power regime did not show any effect. At large laser powers (*P* > 5 mW or power density 7.1 MW cm^−2^ at *w*_0_) an illuminated microparticle suffered irreversible damage as reported previously.^20,21^ Additionally, a bubble rapidly formed next to the microparticle and grew with ongoing illumination. Once the laser was switched off, the bubble slowly shrank and disappeared.

**Fig. 3:**
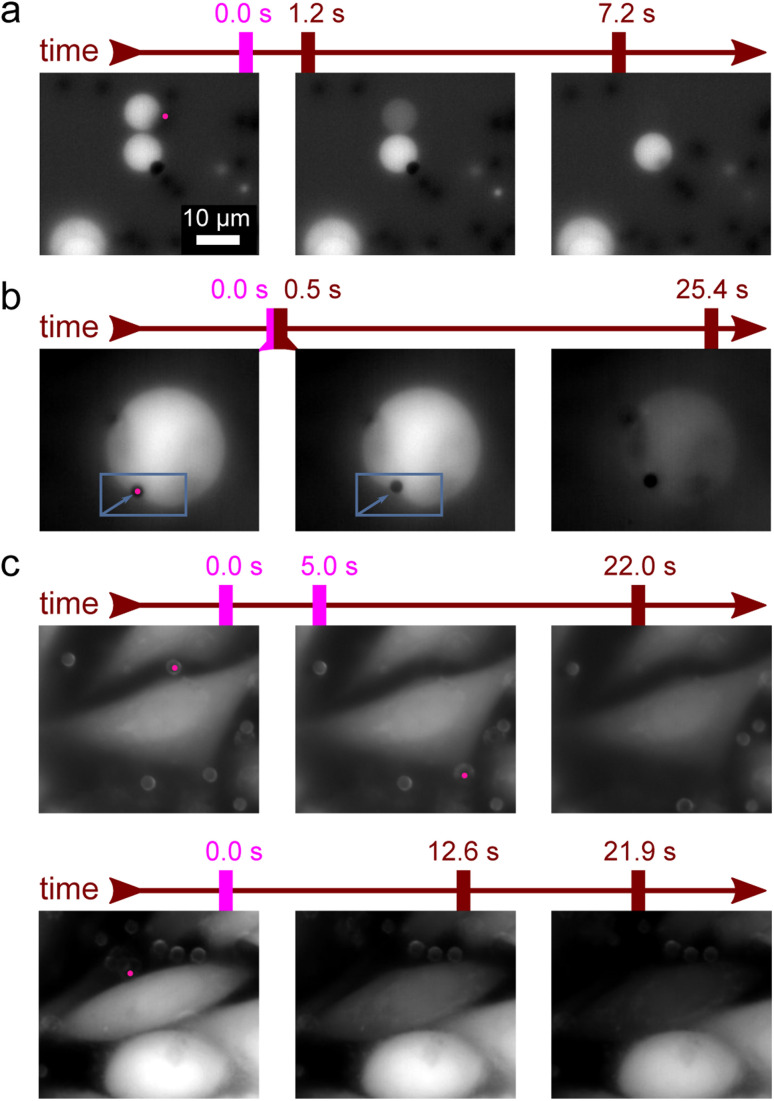
Membrane poration by a photo-acoustic shockwave is a localized phenomena and does not affect living cells. (a) The shockwave perforates only the adjacent vesicle (in this case the top one), while other vesicles remain intact. (b) The shockwave can cause an abrupt local bending of the DPPC membrane (marked by the rectangle and the arrow). (c) Top row: Unaffected cell after two illumination events at low laser power (1 mW< *P* < 5 mW) shows only loss of fluorescence due to photobleaching. Bottom row: a rare example of a cell membrane poration by an exploded microparticle at laser power *P* = 5.2 mW. The scale bar is the same for all images and the pink dot denotes the laser illumination. See also ESI Videos 2–4.

In order to show how these shockwaves can be used as a highly selective method for the local poration of GUVs, we routinely initiated cargo release selectively from only one GUV in the group of several adjacent GUVs (example in Fig. 3a and ESI Video 2†). For large GUV's the shockwave sometimes locally deformed the membrane (example in Fig. 3b and ESI Video 3†).

An important question for the viability of the new method is how it affects living cells. We therefore induced shockwaves in the vicinity of HUVEC cells. Prior to the experiments, the cells were labelled with the fluorescent marker calcein AM. We bound the microparticles to cells and induced shockwaves by optical tweezers as in GUVs. No effects on the cells were observed at laser power below 5 mW (Fig. 3c top row). The observed slow decrease in fluorescence intensity was independent of laser illumination and was thus attributed to photobleaching. Although the experimental setup did not allow us to verify the long-term viability of the affected cells, no calcein leakage due to apoptosis was observed during the course of the experiments. In the context of cargo delivery applications, this is a desirable outcome because the cargo release trigger does not damage the surrounding cells. In contrast, at laser powers above 5 mW, a small percentage of cells were affected (<1%) as the microparticle ripped off a chunk of the cell membrane and caused a rapid decrease in fluorescence (Fig. 3c bottom row and ESI Video 4†).

## 4 Conclusions

In summary, we have demonstrated a novel method for lipid membrane poration with excellent temporal and spatial control. This technique enables targeted cargo delivery encapsulated within a GUV with a single-cell precision. The approach is readily applicable in investigations of the local effects of drugs on a selected group of cells or on a tissue. This method is well-suited for biological applications as NIR light is only weakly absorbed in water and biological tissues. The use of standard optical microscopy and optical tweezers makes the described approach in reach of most of laboratories. However, the short focal length of high numerical aperture objectives used by optical tweezers prevents *in vivo* experiments deep in the tissue. Finally, the controlled delivery of shockwaves, as described in this study, can be used in various soft matter applications where a quick but limited mechanical impulse is needed, and may prove useful in future investigations of the underlying mechanism of sonoporation.

## Author contributions

G. K. and J. D, designed the research and wrote the paper. G. K. and Š. Z.-J. performed the experiments. G. K. analyzed the data. B. M. provided expertise on light–particle interaction. All authors commented on the manuscript.

## Conflicts of interest

There are no conflicts to declare.

## Supplementary Material

RA-013-D3RA03988A-s001

RA-013-D3RA03988A-s002

RA-013-D3RA03988A-s003

RA-013-D3RA03988A-s004

RA-013-D3RA03988A-s005
