# Supplementary material for: Locally induced shockwaves for selective perforation of cargo loaded lipid vesicles with temporal and spatial control
Source: RSC Adv. 2023 Aug 21;13(35):24830–4. doi: 10.1039/d3ra03988a (PMC10440727; doi:10.1039/d3ra03988a)
Supplement: RA-013-D3RA03988A-s001 [file RA-013-D3RA03988A-s001.pdf]

Electronic Supplementary Material (ESI) for RSC Advances.
This journal is © The Royal Society of Chemistry 2023

Supplementary Material for "Locally induced shockwaves for selective perforation of cargo loaded lipid vesicles with temporal and spatial control"

Jure Derganc,[1] Špela Zemljič-Jokhadar,[1] Boris Majaron,[2,3] and Gašper Kokot[1,3]

[1] *Institute of Biophysics, Faculty of Medicine, University of Ljubljana, Ljubljana, Slovenia.*
[2] *University of Ljubljana, Faculty of Mathematics and Physics, Ljubljana, Slovenia.*
[3] *Jožef Stefan Institute, Ljubljana, Slovenia.*

(Dated: August 8, 2023)

VIDEO CAPTIONS

Video 1: An example of an EPC vesicle burst after laser illumination from Fig. 1d. Laser power $1\,\mathrm{mW} < P < 5\,\mathrm{mW}$. The video runs three times faster than real time.

Video 2: A demonstration of localized DPPC vesicle perforation after laser illumination from Fig. 3a. Laser power $1\,\mathrm{mW} < P < 5\,\mathrm{mW}$. The video runs ten times faster than real time.

Video 3: A demonstration of DPPC vesicle membrane bending after laser illumination from Fig. 3b. Laser power $1\,\mathrm{mW} < P < 5\,\mathrm{mW}$. The video runs ten times faster than real time.

Video 4: A demonstration of a rare event of cell membrane poration by an exploded microparticle from Fig. 3c bottom row. Laser power $P > 5\,\mathrm{mW}$. The video runs twenty times faster than real time.

FOCUSED LASER BEAM ILLUMINATION OF A LIGHT ABSORBING MICROPARTICLE

When we illuminate an optically opaque microparticle with a focused laser beam (optical tweezers), the basic response for an unbound particle whose index of refraction is larger than the index of refraction for the surrounding medium, is attraction to the laser focal point. In our case the particle is lateraly attracted to the laser focus and simultaneously pushed away from the focus in the vertical direction. This vertical relocation of the microparticle is seen as a blurring in the brightfield optical images (Fig. S1) and is caused by strong enough photonic pressure (the light propagates from under as indicated in Fig. S1) to overwhelm gravity. In a more standard, optical trapping (or optical levitation) experiment the microparticle is chosen such that the force arrising from photonic pressure balances gravity - this is not our case. For our method to work the particle needs to be in focus long enough to trigger the photo-acoustic effect (unbound particles get pushed out of focus too fast and no shockwave is produced). We experimentally approach this in two ways. One way is to find a microparticle that is non-specifically adhered to the glass substrate. We used this approach in main text Fig. 1a and Fig. 2. These types of experiments regularly release the microparticle from the substrate after the shockwave is triggered (as in Fig. S1 and Fig. 1a). The second way is to bind the microparticle to the GUV. In this case the optical pressure needs to lift both the microparticle and the GUV together, which experience a larger gravitational force and the particle stays in the laser focal point sufficiently to trigger the photo-acoustic effect.

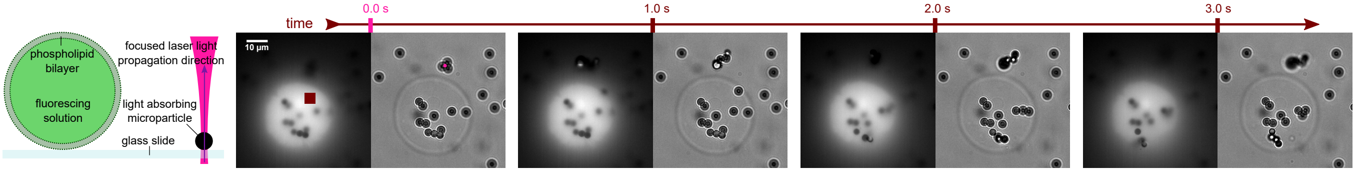

Fig. S1. This is a version of Fig. 1a from the main text, where the whole giant unillamelar vesicle (GUV) is visible. In this particular example the GUV is covered with light absorbing microparticles also in the central region, therefore, we mark with a red square the region we use to demonstrate changes in fluorescence. Pink dot denotes the laser illumination point.
